# Self-perceived health and functional status of older people: Telephone-based lifestyle survey of older adults in Tehran province

**DOI:** 10.34172/hpp.2022.06

**Published:** 2022-05-29

**Authors:** Masoumeh Sadeghipour Rousari, Moloud Payab, Sahar Keyvanloo Shahrestanaki, Mahbube Ebrahimpur, Neda Mehrdad, Solmaz Sadat Naghavi Alhosseini, Faranak Bidmeshgipour, Hossein Adibi, Amirali Safari Astaraei, Raziye Sadat Hosseini, Bagher Larijani, Farshad Sharifi

**Affiliations:** ^1^Public Health Department, School of Public Health and Safety, Shahid Beheshti University of Medical Sciences, Tehran, Iran; ^2^Endocrinology and Metabolism Research Center, Endocrinology and Metabolism Clinical Sciences Institute, Tehran University of Medical Sciences, Tehran, Iran; ^3^School of Nursing and Midwifery, Iran University of Medical Sciences, Tehran, Iran; ^4^Diabetes Research Center, Endocrinology and Metabolism Clinical Sciences Institute, Tehran University of Medical Sciences, Tehran, Iran; ^5^Nursing Care Research Center, Iran University of Medical Sciences, Tehran, Iran; ^6^Idea Development and Innovation Center, Endocrinology and Metabolism Research Institute, Tehran University of Medical Sciences, Tehran, Iran; ^7^Evidence Based Medicine Research Center, Endocrinology and Metabolism Clinical Sciences Institute, Tehran University of Medical Sciences, Tehran, Iran; ^8^Yas Diabetes and Metabolic Diseases Research Center, Tehran, Iran; ^9^Obesity and Eating Habits Research Center, Endocrinology and Metabolism Molecular-Cellular Sciences Institute, Tehran University of Medical Sciences, Tehran, Iran; ^10^Software Developer, Tehran, Iran; ^11^Department of Public Health Nursing and Geriatric, School of Nursing and Midwifery, Iran University of Medical Sciences, Tehran, Iran; ^12^Elderly Health Research Center, Endocrinology and Metabolism Population Sciences Institute, Tehran University of Medical Sciences, Tehran, Iran

**Keywords:** Activities of daily living (ADL), Chronic disease, Incontinence (urinary, fecal), Instrumental Activity of Daily living (IADL), Self-report health

## Abstract

**Background:** The prevalence study of health conditions can help policy makers to document base policymaking. This study aimed to reveal the health status, including the prevalence of geriatric syndrome health conditions such as activity of daily livings, pain, and physical and mental health of older adults in Tehran province.

**Methods:** This cross-sectional study was a telephone survey with older people ≥60 years old using a systematic random sampling of telephone numbers in Tehran province. The Persian version of the Katz’ activity of daily living (ADL) and the Lawton’s instrumental activity of daily living (IADL) questionnaires were used to evaluate the functional status. Pain, history of chronic diseases, continence, hospital admission, sensory problems, and self-perceived health (SPH) were asked by trained nurses or gerontologists thorough telephone interviews.

**Results:** In this study, 1251 older adults with the mean age of 67.03±7.51 years have been recruited. About 64.50% (95% CI: 64.4-64.6) of them were totally independent according to ADL (female=60.02% and male=68.50%), and about 40.50% (95% CI: 40.4-40.5) were independent based on IADL domains (female=39.41% and male=41.80). The dependency rates in ADL increased with the aging of population. Joint pain was the most prevalent type of pains and near to 26.00% (95% CI: 64.4-64.6) of the participants suffered moderate joint pains. About 71.5% (95% CI: 71.4-71.5) of the participants were urinary continent (female=67.66% and male=76.06%), and 91.9% (95% CI: 91.9-92.0) had bowel control (female=91.47% and male=92.94%) and the prevalence of incontinence increased by advancing age. Only 26.70% (95% CI: 26.6-26.8) of the participants reported excellent and good levels of perceived health status (female=21.98% and male=31.48%) and about 26.2% (95% CI: 26.1-26.2) of them reported some degree of visual impairment.

**Conclusion:** The results of the present study can provide a good view about the health profile of older adults, including pain, functional status, sphincter control, chronic diseases, sensory status, and SPH. Future studies should prioritize SPH as an important predictor of mortality rates.

## Introduction

Increasing life expectancy and the decreased fertility rate in communities has led to a significant worldwide growth in the aging population with impressive needs to health care services in recent decades.^1,2^ Although longer living due to the reduced mortality is a valuable achievement of advances in health sciences, it may be accompanied with an increased prevalence of multimorbidities, disabilities, and the incremental increases in health care utilization.^3,4^ Chronic non-communicable diseases are still the most common causes of death and disability in older population, particularly in low and middle income countries.^5,6^ Multimorbidities not only lead to more health care costs, but also will result in poorer subjective well-being.^7,8^

Iran will face a huge number of old population in near future.^9^ The aging population of Iran ( > 60 years) has increased from 6.6% in 1996 to 9.3% in 2016, and the number of old people is expected to double between 2015 and 2030.^10^ Therefore, meeting health care needs of the aged population is a challenging necessity for the health system.^11,12^ Priority setting while allocating the resources needs a comprehensive knowledge about the health status of community dwelling older population.

Knowing the health status, including functional status, disease profile, pain, continence, and sensory status as well as self-perceived health (SPH) can be helpful for health policymakers to design appropriate care and preventive programs for older adults. Moreover, the subjective aspects of health are important in a similar vein to objective ones in the profile of active aging.^13^ SPH is a simple method for evaluating the individual’s health status. SPH is a powerful predictor of mortality and morbidity in old age, and is affected by many different factors such as physical and mental health, and cultural background.^14,15^ Numerous studies have revealed an association between SPH and health outcomes, including functional limitations,^16^ frailty^17^ and mortality^15^ among older people. The socio-cultural structure of communities may affect the health condition of individuals.^18^ There is a lack of evidence regarding the perception of various dimensions of health status in low- and low-middle income countries.^16^

There is a scarcity of data about the health status of older adults in Iran, particularly in Tehran province. Some of these data is very crucial for policymaking, including data of independency in activity of daily living (ADL) and instrumental activity of daily living (IADL), as well as the prevalence of multimorbidity.

This study was designed to assess the epidemiologic characteristic of health condition, independency, multimorbidity, and SPH condition of the older adults in Tehran province. Tehran province is the most populous province of Iran and one-sixth of Iranian older adults are living in this province. Because of huge immigration of population, the population of Tehran province is approximately representative of Iran’s population. This study provided data about self-report health condition and history of health problems in a representative sample of community dwelling older people of Tehran province. As the health indices are significantly different between both sexes in older adults, we need the reliable data on each sex for policymaking. Although similar research is conducted in Tehran province, but the present research is unique in term of scope.^19^ Therefore, this study aimed to evaluate the geriatric syndrome, dependency in activities of daily living, health utilization, physical and mental health status in the mentioned population.

## Material and Methods

### Setting and sampling

This is a cross-sectional telephone-based survey carried out from July 2015 to February 2016. This study had a representative sample of community dwelling older adults who lived in Tehran province which has more than 13 million populations and is the most populous province in Iran encompassing 10.4% of adults aged ≥60 years old. This province has 16 counties; and Tehran metropolitan itself has 22 districts. The sampling of study is proportionated to rural and urban regions. The sampling was based on telephone numbers using a systematic random sampling for each county and each district of Tehran metropolitan. Before the random sampling, the telephone numbers of non-residential places were excluded. As the proportion of aged adults living in Tehran province is about 10% of the total general population and the expected response rate was estimated as 50%, the number of telephone was estimated 20 times greater than the sample size. In cases that there were no older adults in family or if there was an older adult unwilling to participate, the next telephone numbers were replaced to reach to a family that one of members had criteria for enrollment and agree to participate.

In order to decrease intracluster correlation between the data of the samples, in some cases that two or more older adults were living in a residential place (apartment or house), one was randomly selected.

### Participants

Old adults aged ≥ 60 years who could communicate via telephone were included. Those who were excluded from the research were elderlies with moderate to severe cognitive impairment, according to state of their caregivers (which impaired his/ her daily function due to cognitive impairment), patients with end stage cancer, and bed ridden older adults. Those subjects diagnosed with dementia, but had no problem in daily functions were included in the study.

### Data collection

The participants data on demographic characteristics, history of past health conditions, history of hospital admission in the last 6 months and times of admission, and continence (bowel and urine) were collected using a questionnaire approved by an expert panel. Pain assessment was performed by asking the participant about presence, location, and severity of chronic pains. The items for pain evaluation were derived from Brief Pain Inventory (BPI). Sensory status, oral health, sleep status and denture having were asked during the telephone-based interview. ADL and IADL were assessed using the Persian version of Katz’ ADL^20^ and Lawton’s IADL.^21^ Katz’ ADL included 6 items, and a score of 12 indicates full function (each item can change from zero to 2 score) and the minimum score could be zero. The highest score of Lawton’s IADL is 8 indicating the complete independency and the minimum score is zero. SPH was evaluated using the well-known question. We assessed history of hospital admission, falls, and accidents over last six months by asking participants or one of informants. Self-reported history of health problems such as diabetes, hypertension, dyslipidemia, coronary heart disease, cerebrovascular disease, thyroid problems, peripheral vascular disease, heart failure, renal failure, liver failure, dementia, Parkinson’s, chronic obstructive pulmonary disease, cancers, infections, including pneumonia and urinary tract infections last year, and the history of aging syndromes such as delirium, pressure ulcers, urinary and feces incontinence in the last 6 months, were revealed by asking from the participants or their informants. The drug history and previous health conditions were self-reported and answered by the study participants themselves.

Trained nurses or gerontologists performed the interviews by telephone during one or two call sessions. Each interview lasted between 20 to 40 minutes. In cases of doubtful responses, interviews were performed with one of the participants’ informants.

Suffering from any type of pain, locations and severity of pain also interfering of pain with the work were detected in the interviews.

The data about lifestyle is based on a questionnaire created by researchers that was inspired by two questionnaires, HELP and Life Style Profile II, and includes questions about seven categories, including physical activity, smoking, alcohol consumption, and sleep characteristics.

The data collected through the telephone survey by the researchers were recorded in the ASP.NET MVC web application and stored in the MSSQL database.

### Statistical analysis

The statistic significant were defined as *P* values < 0.05. All statistical tests were performed as two-way. As the prevalence rates of the conditions that we reported are population-structure related, we carried out as survey analysis with weights equal to the inverse of the sample-population ratio to address this concern. Description of the prevalence was conducted by the point estimation of frequency and 95% confidence interval. Normal distributed variables were compared using independent *t* test. The proportions were compared using χ2. All analysis were done using STATA package version 11.0 (Stata Statistical Software: Release 11. StataCorp LP. Package, College Station, TX, USA).

## Results

In this study, 1251 aged people (54.83% were female) participated with the mean age of 67.03 ± 7.51 years. In total, 74.82% (936) of participants were married, and 96.91% of them had children. (See Table 1).

**Table 1:** The characteristics of the participants

**Variable**		**Female (n=686)**	**Male (n=565)**	**Total (N=1251)**	***P*** ** value**
Age groups (%) (N = 1251)	60-69 y	453 (66.03)	334 (59.12)	787 (62.91)	0.040
	70-79 y	176 (25.66)	177 (31.33)	353 (28.22)	
	≥80 y	57 (8.31)	54 (9.56)	111 (8.87)	
Marital status (%) (N = 1251)	Married	420 (61.22)	516 (91.33)	936 (74.82)	< 0.001
	Single	13 (1.9)	9 (1.59)	22 (1.76)	
	Widow	232 (33.82)	23 (4.07)	255 (20.38)	
	Divorce	11 (1.6)	1 (0.18)	12 (0.96)	
	Separated	5 (0.73)	5 (0.88)	10 (0.8)	
	Others	5 (0.73)	11 (1.95)	16 (1.28)	
Education level (%) (N = 1251)	Illiterate	211 (30.76)	76 (13.45)	287 (22.94)	<0.001
	Primary school	231 (33.67)	146 (25.84)	377 (30.14)	
	High school	77 (11.22)	115 (20.35)	192 (15.35)	
	Diploma	103 (15.01)	115 (20.35)	218 (17.43)	
	Academic education	64 (9.33)	113 (20)	177 (14.15)	
Career status (%) (N = 1251)	Unemployed	50 (7.29)	73 (12.92)	123 (9.83)	< 0.001
	Retired	93 (13.56)	330 (58.41)	423 (33.81)	
	Employed after retirement	9 (1.31)	87 (15.40)	96 (7.67)	
	Employed	25 (3.64)	69 (12.21)	94 (7.51)	
	Housekeeper	509 (74.2)	6 (1.06)	515 (41.17)	
Smoking (%) (N = 1251)	Never	655 (95.48)	444 (78.58)	1099 (87.85)	< 0.001
	1 Day per week	12 (1.75)	22 (3.89)	34 (2.72)	
	2 Days per week	5 (0.73)	27 (4.78)	32 (2.56)	
	3 Days per week	5 (0.73)	9 (1.59)	14 (1.12)	
	4 Days or more per week	9 (1.31)	63 (11.15)	72 (5.76)	
Alcohol consumption (%) (N = 1251)	Never	668 (97.38)	541 (95.75)	1209 (96.64)	0.113
	1 Day per week	4 (0.58)	12 (2.12)	16 (1.28)	
	2 Days per week	5 (0.73)	7 (1.24)	12 (0.96)	
	3 Days per week	4 (0.58)	3 (0.53)	7 (0.56)	
	4 Days or more per week	5 (0.73)	2 (0.35)	7 (0.56)	
Physical activity (%) (N = 1251)	First quartile	204 (29.74)	99 (16.28)	296 (23.66)	< 0.001
	Second quartile	149 (21.72)	112 (19.82)	261 (20.86)	
	Third quartile	167 (24.34)	195 (34.51)	362 (28.94)	
	Fourth quartile	166 (24.2)	166 (29.38)	332 (26.54)	

Qualitative variables were reported as numbers and percentages. The Chi-square test was used to analyze qualitative variables.

### Functional status

Table 2 shows the functional status of the participants defined by the ADL (Katz) and IADL (Lawton) in different gender and age groups. More than 42.0% of the female > 80 years were totally dependent while, 42.5% of male > 80 years were totally independent by ADL. Near to 73.6% of female and 55.5% of male > 80years were dependent in 3 domain or more by IADL.

**Table 2:** Functional status (ADL KATZ and IADL Lawton scores) of the participants in age groups

		**Age groups (y)**		
	**Gender**	**60-69 (n=787)**	**70-79 (n=353)**	**≥80 (n=111)**	**Total N=1251**
**ADL KATZ (N=1251)**					
Totally dependent 95% CI	Female	7.2 (7.2-7.3)	9.6 (9.5-9.7)	42.1 (41.8-42.3)	12.9 (12.8-12.9)
	Male	8.3 (8.3-8.4)	15.2 (15.1-15.3)	31.4 (31.2-31.7)	
Partially dependent 95% CI	Female	23.8 (23.7-23.9)	34.6 (34.5-34.8)	26.3 (26.0-26.5)	22.5 (22.4-22.5)
	Male	12.5 (12.4-12.6)	24.8 (24.7-24.9)	25.9 (25.6-26.1)	
Totally independent 95% CI	Female	68.8 (68.7-68.9)	55.6 (55.5-55.8)	31.5 (31.3-31.8)	64.5 (64.4-64.6)
	Male	79.0 (78.9-79.1)	59.8 (59.7-60.0)	42.5 (42.3-42.8)	
**Lawton IADL score (N=1251)**					
Independent	Female	52.5 (52.4-52.6)	25.0 (24.8-25.1)	10.5 (10.3-10.7)	40.5 (40.4-40.5)
	Male	52.9 (52.8-53.1)	32.7 (32.6-32.9)	11.1 (10.9-11.2)	
One domain dependent	Female	20.9 (20.8-21.0)	19.8 (19.7-20.0)	8.7 (8.6-8.9)	19.9 (19.8-19.9)
	Male	20.0 (19.9-20.1)	21.4 (21.3-21.6)	22.2 (21.9-22.4)	
Two domains dependent	Female	9.7 (9.6-9.7)	21.0 (20.8-21.1)	7.0 (6.8-7.1)	12.15 (12.11-12.19)
	Male	10.1 (10.1-10.2)	15.2 (15.1-15.3)	11.1 (10.9-11.2)	
Three domains or more dependent	Female	16.7 (16.7-16.8)	34.0 (33.9-34.2)	73.6 (73.4-73.9)	27.4 (27.3-27.4)
	Male	16.7 (16.6-16.8)	30.5 (30.3-30.6)	55.5 (55.2-55.8)	

Abbreviations: ADL, Katz Index of Independence in Activities of Daily Living; CI, confidence interval; IADL, The Lawton Instrumental Activities of Daily Living Scale.

### Pain

Among all the participants, 79.4% (95% CI = 79.4-79.5) mentioned that they were suffering from any pain that 87.8% (95% CI = 87.7-87.8) of women and 71% (95% CI = 70.9-71.1) of men reported to have pain at a least one organ. However, by gender, 82.7% (95% CI = 82.6-82.8) of females with older ages were experiencing pain and this portion seems to increase with age so that most age groups experiencing pains were women older than 80 years old. (91.2%; 95% CI = 91.1-791.4).

Among the participants, 26.0% (95% CI = 26.0-26.1) asserted that they suffered moderatejoint pain and 20.5% (95% CI: 20.5-20.6) of them had history of mild headache. The prevalence of sever joint pain was more common in the age-group of > 80 years old (35%; 95% CI = 34.8-35.3) (see Supplementary file 1, Table S1).

### Pain effect 

Among participants, 4.2% (95% CI: 4.1-4.2) of the responders stated that “their pain has extremely interfere with normal work” and 23.7% (95% CI: 23.6-23.8) stated that “quite a bit interferes with their work”, and the highest proportion of the participants; 34.7% (95% CI: 34.6-34.8) reported the pain moderately interfered with their normal work; 28.3% (95% CI: 28.2-28.3) reported the pain interference was “a little bit” and only 8.8% (95% CI: 8.8-8.9) reported that they have no pain “not at all” (See Table S2).

### Self-perceived health 

Only 13.01% (95% CI: 12.97-13.05) of the participants reported their perceived health reported as excellent, 59.90% (95% CI: 59.85-59.96) of the participants reported that had very good health condition, and 5.00% (95% CI: 4.97-5.02) reported as suffering bad health condition.

### Balance problems

Totally, 65.4 % (95% CI: 65.4-65.5) of older adults reported that they had balance problems. The highest prevalence of balance problem was observed in women in the age-group ≥80 years (47.3%; 95% CI 47.0-47.6) (See Table S3).

Amongst the participants, 11.6% (95% CI: 11. 5-11.6) had a history of hospital admission over the last 6 months, and the age group of 70-79 years had the highest proportion of hospital admission among all age groups. The detailed information about the hospital admission times in all age groups and the disease profile of the participants are listed in Table S4.

### Medical history

Totally 57.5% (95% CI: 57.4-57.5) of older adults reported that they had ≥ 3 comorbidities. The most prevalent reported health problem among the participants was osteoarthritis 46.1% (95% CI: 46.0-46.1). Moreover, 40.6% (95% CI: 40.5-40.6) of older adults had a history of hyperlipidemia (see Table S5, S6).

### Using assistive instruments

Of total, 64.72% (95% CI: 64.66 – 64.77) and 8.66% (95% CI: 8.63 – 8.70) of participants were using glasses and hearing aid, respectively (see Table S7)

### Teeth and eating ability

Among the participants, 60.2% (95% CI: 60.1-60.2) were using dentures. Also 90.3% (95% CI: 90.2-90.3) had intact eating ability (see Table S8).

### Sleep status

Of total, 32.7% (95% CI: 32.6-32.8) had problems with sleep, 40.3% (95% CI: 40.0-40.6) female participants aged > 80 years had problem with sleep (see Table S9).

### Continence

Amongst the participants, 71.5% of them (95% CI: 71.4-71.5) stated that they could completely control their urine sphincters, and 5.3% reported that was completely incontinent. In addition, 91.9% of them (95% CI: 91.9-92.0) stated that they could completely control their stool sphincter, and 3.1% reported that have fecal incontinence (see Table S10).

## Discussion

This study was carried out to assess geriatric syndrome, functional status (ADL and IADL), health conditions such as pain, polypharmacy, sensory disability, incontinence, and SPH status of older adults in Tehran province. Despite the heterogeneity of older adults, it seems that with the aging of population, a poorer health situation among older adults was expected which may require more comprehensive health service packages.

### Functional status

Near to two third of the participants were totally independent in terms of ADL and IADL and this rate increased with aging of the population; More than one third of ≥80 years old group, while less than one tenth of participants aged 60-69 years were dependent in ADL. In general, more than one third of the community dwelling older adults in all age groups needed some degree of helping for performing of ADL. This high proportion of dependency in this Iranian older population should be considered by the policy makers to extend elderly home care services. Furthermore, only a little more than 40% of the participants were able to do their chores in IADL independently. As the population of older adults is growing in Iran with the changes in the family structures, it is expected that the responsibility of care for older adults will be moved from family members to the formal care settings during in the near future. Although Taheri Tanjani et al reported an independency of 86% in ADL among older adults in a sample from 5 provinces of the country, as the participants of that study were somehow active members of health care centers, they may underestimate the dependency prevalence among the older adults because their participants had a better functional status than the target population. On the other hand, five-province in 2012 over Iran reported that only 31% of the participants were independent in IADL; which was a little less than our study. This discrepancy may be due to this fact that they evaluated less than 8 domains in study, while we used standard version of IADL questionnaire (8 domains).^22^

### Pain

Suffering from any chronic pain was reported in near four fifth of the participants, and we observed the highest rate among the age group 70 to 79 years old, which is almost similar to the study of Zarei et al in the South of Iran (76% in over 70 years old population).^23^ The most common reported sites of chronic pain in our study were joints, which Zarei et al has also reported joint pain as the most prevalent pain site (foot and joint pain had the share of 31.9%).

As chronic pain is a common debilitating complain in older adults, which affects mental health,^24^ physical, and social activity also imposes extra costs on health system,^24,25^ it seems that the detection and management of chronic pain should be a priority of the primary health care system for older adults, and can effectively improves different dimensions of older adults’ health and decreases the refers to physicians, as well.

### Self-perceived health

Most of the participants reported optimal self- perceived health (excellent, good and fair), which is comparable with the findings of Ghalichi et al (about 70% in the group of > 65 years)^26^ and the study of Mirzaie (57.5% good, 5.1% excellent and 8.8 % very good),^27^ despite their different method of categorization. Beside all other factors, SPH is deeply dependent to culture. It looks like that Iranian older people less intersected in reporting their health condition as ‘‘excellent’’ or ‘‘very good’’.^28^ Therefore, most of the participants reported fair health (about 60%) instead of good or excellent. According to the results, SPH is deteriorated by older age as the poor health category changes from about 8% in 60–69 years old to about 32% among equal or over 80 years old adults. This deterioration can be due to the deterioration in physical health and functional status. In addition, about 45% of the participants reported improvement in their health status in comparison with the previous year and just about 20% of them estimated their health condition became worse. As self- perceived health is an important context-based variable, which can predict mortality rate^14^ in older adults, further studies maybe need to evaluate the factors that affect the perceived health status included depression and mood condition.^29^

### Urine and fecal continence

Among the participants, urinary incontinence (UI) reported in more than one fifth of the participants reported occasional UI, and less than 4% of them reported no had control. The prevalence of UI increased by age, so that in the age group ≥80 years, more than 40% were suffering from occasional or sustain UI. However, the prevalence of UI was reported more than 60% in older women in Yazd. As culturally, incontinence is assumed as a normal age-related change and older people think that there is no an effective treatment for it, they don’t seek medical treatment and try to cope with this problem, which is perceived as a matter of shame in their view.^30^ It imposes not only a huge costs on society, but also is associated with depression and mortality.^31^

Fecal incontinence is also another embarrassing feeling, which is called as “silent suffering”.^32^ In agreement with the reported prevalence in the community based studies^33^ near to 8% of the participants reported fecal incontinence(occasional or general), while in Iran Alimohammadian et al reported 18.4% FI in over 40 years older women.^32^ It seems that UI is usually unrecognized by primary health physicians, whereas there are a lot of noninvasive interventions to improve incontinence in older adults.^34^ Applying screening programs for both urine and stool incontinence in primary care settings can be helpful in detecting and treating of incontinence and ultimately improves the quality of life among this age group.

### Visual and hearing

While, the prevalence of blindness has been reported in 4% in a systematic review on visual problems of people over 50 years in Iran,^35^ in the present research, it was reported in less than 0.5% of the participants, which could be because of less participation of blind people in this study. Although less than 30% of the participants reported a kind of hearing impairment and only less than 10% of them used hearing aid. Despite the high costs of hearing aid and lack of knowledge among older people and their family members, it seems that there is not cultural acceptance for hearing aids in Iranian older adults.

We used telephone survey with a stratified simple random sampling based on the restricts of Tehran metropolitan and all cities exist in Tehran province. This method is a known valid method for choosing a representative population for enrollment into studies.^36^

### Strengths and limitations

Recruiting sufficient participants through random sampling, which consisted population of all counties and districts of Tehran province, and the acceptable response rate (about 90%) was the strength point of this study. However, as the study was a telephone based survey, and face to face interview was not possible, most of the collected data were based on the judgment of older adults and sometimes may be biased, despite the quest’s effort. Moreover, the end stage older adults who were not able to communicate were excluded. Therefore, the functional status and health profile may be estimated a bit better than the real situation.

## Conclusion

The results of the present study can provide a good view about the health profile of older adults, including pain, functional status, sphincter control, chronic diseases, sensory status, and SPH. Since the proportion of the elderly over the age of 60 is rising, the task of caring for this population will most likely be transferred from family members to government care facilities for the elderly in the near future. Given this, governments must accurately predict the aging populous necessities. One of the results of this study was the high incidence of chronic pain among the elderly, which, as it impairs their quality of life, necessitates attention to this point among these people’s relevant physicians. Future studies should prioritize SPH as an important predictor of mortality rates. Urinary or fecal incontinence should be included in screening programs for older adults. This is a telephone-based study that, despite its flaws, will assist researchers and policymakers in future planning for care for older adults in the Metropolitan province.

## Acknowledgements

The authors would like to acknowledge all of the older adults who participants in this research

## Authors’ contributions

MSR, NM, and FSH participated in the study design, final revision and edit. MP, SK, AND ME participated in the sequence alignment and draft the manuscript. SN, FB, and RH participated in the data acquisition. FSH and HA performed statistical analysis. BL participated in critical review. All authors read and approved the final manuscript.

## Funding

This work was financially supported by Tehran University of Medical Sciences.

## Ethical approval

Verbal informed consent was obtained via telephone interview. The ethical approval was taken from the Ethical Research Committee of Tehran University of Medical Sciences (TUMS) (ethical code: 26572).

## Competing interests

The authors declare that they have no conflict of interest.

## Supplementary Materials

Supplementary file 1 contains Tables S1-S10.
