# Supplementary material for: Self-perceived health and functional status of older people: Telephone-based lifestyle survey of older adults in Tehran province
Source: Health Promot Perspect. 2022 May 29;12(1):37–44. doi: 10.34172/hpp.2022.06 (PMC9277287; doi:10.34172/hpp.2022.06)
Supplement: Supplementary file 1 — contains Tables S1-S10. [file hpp-12-37-s001.pdf]

Supplementary file 1

Table S1. Locations of chronic pain in age groups

			Age groups			
**Pain location**	**severity**	**Gender**	**60 – 69 years N = 787 (%)**	**70 – 79 years N = 353 (%)**	**≥80 years N =111 (%)**	**Total N = 1251**
**Joint pain** (95%CI)	no pain	Female	30.0(29.9-30.1)	25.0(24.8-25.1)	19.2(19.0-19.5)	35.4(35.3-35.4)
		Male	46.4(46.2-46.5)	33.3(33.1-33.4)	53.7(53.4-53.9)	
	mild pain	Female	19.4(19.3-19.5)	13.0(12.9-13.1)	10.5(10.3-10.7)	16.9(16.9-17.0)
		Male	17.6(17.5-17.7)	20.9(20.7-21.0)	9.2(9.0-9.4)	
	moderate pain	Female	26.9(26.8-27.0)	31.8(31.6-31.9)	31.5(31.3-31.8)	26.0(26.0-26.1)
		Male	23.0(22.9-23.1)	29.9(29.7-30.0)	9.2(9.0-9.4)	
	severe pain	Female	20.0(20.0-20.1)	23.2(23.1-23.4)	35.0(34.8-35.3)	18.0(17.9-18.0)
		Male	10.4(10.4-10.5)	14.1(14.0-14.2)	22.2(21.9-22.4)	
	very severe pain	Female	3.5(3.4-3.5)	6.8(6.7-6.8)	3.5(3.4-3.6)	3.53(3.51-3.55)
		Male	2.3(2.3-2.4)	1.6(1.6-1.7)	5.5(5.4-5.6)	
**Headache (95%CI)**	No pain	Female	61.3(61.2-61.4)	61.9(61.7-62.0)	78.9(78.7-79.1)	65.5(65.5-65.6)
		Male	67.0(66.9-67.1)	64.9(64.8-65.1)	74.0(73.8-74.3)	
	Mild pain	Female	21.1(21.1-21.2)	21.5(21.4-21.7)	5.2(5.1-5.3)	20.5(20.5-20.6)
		Male	21.5(21.4-21.6)	25.9(25.8-26.1)	14.8(14.6-15.0)	
	Moderate pain	Female	11.6(11.6-11.7)	11.3(11.2-11.4)	12.2(12.0-12.4)	9.33(9.30-9.37)
		Male	8.3(8.3-8.4)	5.6(5.5-5.7)	3.7(3.5-3.8)	
	Severe pain	Female	5.0(5.0-5.1)	3.4(3.3-3.4)	3.5(3.4-3.6)	3.7(3.6-3.7)
		Male	2.9(2.9-3.0)	2.8(2.7-2.8)	3.7(3.5-3.8)	
	Very severe pain	Female	0.66(0.64-0.067)	1.7(1.6-1.7)	0[1]	0.75(0.74-0.76)
		Male	0	0.56(0.54-0.58)	3.7(3.5-3.8)	
**Back pain (95%CI)**	no pain	Female	43.9(43.8-44.0)	45.4(45.2-45.6)	49.1(48.8-49.4)	51.5(51.4-51.5)
		Male	57.4(57.3-57.5)	52.5(52.3-52.7)	72.2(71.9-72.4)	

[1] no observations

	mild pain	Female	20.3(20.2-20.3)	13.0(12.9-13.1)	15.7(15.5-15.9)	18.0(18.0-18.1)
		Male	17.3(17.2-17.4)	23.1(23.0-23.2)	12.9(12.7-13.1)	
	moderate pain	Female	22.0(21.9-22.1)	22.7(22.5-22.8)	17.5(17.3-17.7)	17.9(17.8-17.9)
		Male	15.2(15.1-15.3)	14.1(14.0-14.2)	9.2(9.0-9.4)	
	Severe pain	Female	10.8(10.7-10.8)	14.7(14.6-14.8)	17.5(17.3-17.7)	10.2(10.1-10.2)
		Male	8.3(8.3-8.4)	7.9(7.8-7.9)	3.7(3.5-3.8)	
	Very severe pain	Female	2.8(2.8-2.9)	3.9(3.9-4.0)	0	2.2(2.2-2.3)
		Male	1.4(1.4-1.5)	2.2(2.2-2.3)	1.8(1.7-1.9)	
**Neck pain (95%CI)**	no pain	Female	56.0(55.9-56.1)	61.9(61.7-62.0)	75.4(75.1-75.6)	64.6(64.5-64.7)
		Male	67.0(66.9-67.1)	68.3(68.2-68.5)	79.6(79.3-79.8)	
	mild pain	Female	22.2(22.2-22.3)	18.1(18.0-18.3)	3.5(3.4-3.6)	18.1(18.0-18.1)
		Male	19.4(19.3-19.5)	17.5(17.3-17.6)	9.2(9.0-9.4)	
	moderate pain	Female	11.6(11.6-11.7)	12.5(12.3-12.6)	10.5(10.3-10.7)	10.4(10.4-10.5)
		Male	9.8(9.8-9.9)	10.1(10.0-10.2)	3.7(3.5-3.8)	
	Severe pain	Female	8.1(8.1-8.2)	5.6(5.6-5.7)	10.5(10.3-10.7)	5.53(55.0-55.6)
		Male	3.2(3.2-3.3)	2.2(2.2-2.3)	5.5(5.4-5.6)	
	Very severe pain	Female	1.7(1.7-1.7)	1.7(1.6-1.7)	0	1.2(1.1-1.2)
		Male	0.02(0.02-0.03)	1.6(1.6-1.7)	1.8(1.7-1.9)	
**Abdominal pain (95%CI)**	no pain	Female	67.7(67.6-67.8)	65.9(65.7-66.0)	85.9(85.7-86.1)	71.2(71.1-71.2)
		Male	72.7(72.6-72.8)	70.0(69.9-70.2)	79.6(79.3-79.8)	
	mild pain	Female	21.6(21.5-21.7)	23.2(23.1-23.4)	1.7(1.6-1.8)	19.3(19.2-19.3)
		Male	20.9(20.8-21.0)	22.5(22.4-22.7)	3.7(3.5-3.8)	
	moderate pain	Female	6.6(6.5-6.6)	6.8(6.7-6.8)	10.5(10.3-10.7)	5.9(5.9-6.0)
		Male	3.5(3.5-3.6)	5.0(5.0-5.1)	9.2(9.0-9.4)	
	Severe pain	Female	2.8(2.8-2.9)	3.9(3.9-4.0)	1.7(1.6-1.8)	2.73(27.1-27.5)

		Male	2.0(2.0-2.1)	1.6(1.6-1.7)	5.5(5.4-5.6)	
	Very severe pain	Female	1.1(1.0-1.1)	0	0	0.7(0.6-0.7)
		Male	0.05(0.05-0.06)	0.056(0.054-0.058)	1.8(1.7-1.9)	
**Other locations (95%CI)**	no pain	Female	59.1(59.0-59.2)	59.0(58.9-59.2)	66.6(66.4-66.9)	60.9(60.9-61.0)
		Male	62.5(62.4-62.6)	58.1(58.0-58.3)	66.6(66.3-66.9)	
	mild pain	Female	20.7(20.6-20.8)	14.7(14.6-14.8)	5.2(5.1-5.3)	18.3(18.2-18.3)
		Male	20.0(19.9-20.1)	22.5(22.4-22.7)	11.1(10.9-11.2)	
	moderate pain	Female	11.4(11.4-11.5)	15.3(15.2-15.4)	12.2(12.0-12.4)	11.6(11.5-11.6)
		Male	10.1(10.1-10.2)	13.5(13.4-13.6)	5.5(5.4-5.6)	
	Severe pain	Female	7.0(7.0-7.1)	7.3(7.3-7.4)	15.7(15.5-15.9)	7.43(7.40-7.47)
		Male	6.5(6.5-6.6)	4.5(4.4-4.5)	11.1(10.9-11.2)	
	Very severe pain	Female	1.54(1.52-1.57)	3.4(3.3-3.4)	0	1.63(1.61-1.65)
		Male	0.05(0.05-0.06)	1.1(1.0-1.1)	5.5(5.4-5.6)	

Table S2. Pain effect in age and sex groups

**Pain effect**			**Age groups**			**total**
	**effect**	**Gender**	**60 – 69 years N = 787 (%)**	**70 – 79 years N = 353 (%)**	**≥80 years N = 111 (%)**	**Total N=1251**
	extremely	Female	2.4(2.3-2.4)	3.9(3.9-4.0)	14.0(13.8-14.2)	3.5(3.5-3.6)
		Male	1.4(1.4-1.5)	1.6(1.6-1.7)	11.1(10.9-11.2)	

	quite a bit	Female	20.9(20.8-21.0)	27.8(27.6-27.9)	42.1(41.8-42.3)	20.0(19.9-20.0)
		Male	11.6(11.6-11.7)	18.0(17.9-18.2)	18.5(18.2-18.7)	
	moderately	Female	36.2(36.1-36.2)	35.7(35.6-35.9)	28.0(27.8-28.3)	33.1(33.0-33.1)
		Male	31.4(31.3-31.5)	30.5(30.3-30.6)	31.4(31.2-31.7)	
	a little of bite	Female	29.1(29.0-29.2)	22.1(22.0-22.2)	14.0(13.8-14.2)	29.6(29.5-29.6)
		Male	34.4(34.3-34.5)	34.4(34.3-34.6)	31.4(31.2-31.7)	
	not at all	Female	11.2(11.1-11.3)	10.2(10.1-10.3)	1.7(1.6-1.8)	13.6(13.5-13.6)
		Male	20.9(20.8-21.0)	15.2(15.1-15.3)	7.4(7.2-7.5)	

Table S3. Balance Problems in age and sex groups

		**Age groups**			
**Balance Problems**	**Gender**	**60 – 69 years<br>N = 787<br>(%)**	**70 – 79 years<br>N = 353<br>(%)**	**≥80 years<br>N = 111<br>(%)**	**Total<br>N = 1251**
	Female	37.7(37.6-37.8)	35.2(35.0-35.3)	47.3(47.0-47.6)	34.5(34.4-34.5)
	Male	26.9(26.8-27.0)	35.5(35.4-35.7)	37.0(36.7-37.3)	
**Balance Standing**	Female	24.7(24.6-24.8)	26.1(25.9-26.2)	38.5(38.3-38.8)	24.0(24.0-24.1)
	Male	17.0(16.9-17.1)	28.2(28.1-28.3)	24.0(23.8-24.3)	

**Balance Standing**	Female	3.9(3.9-4.0)	3.9(3.9-4.0)	7.0(6.8-7.1)	4.25(4.23-42.8)
	Male	2.3(2.3-2.4)	6.2(6.1-6.2)	7.4(7.2-7.5)	

Table S4. The hospital admission times in age and sex groups of the participants during the previous 6 months.

		**Age groups**			
**Hospital admission times**	**Gender**	**60 – 69 years N = 787 (%)**	**70 – 79 years N = 353 (%)**	**≥80 years N = 111 (%)**	**Total N = 1251**
**No hospital admission**	Female	86.5(86.4-86.6)	80.6(80.5-80.8)	84.2(84.0-84.4)	82.8(82.7-82.8)
	Male	83.5(83.4-83.6)	75.7(75.5-75.8)	81.4(81.2-81.7)	
**Once**	Female	9.7(9.6-9.7)	12.5(12.3-12.6)	8.7(8.6-8.9)	11.6(11.5-11.6)
	Male	10.7(10.7-10.8)	16.9(16.8-17.0)	12.9(12.7-13.1)	
**Twice or more**	Female	3.75(3.71-3.79)	6.8(6.7-6.8)	7.0(6.8-7.1)	5.5(5.5-5.6)
	Male	5.6(5.6-5.7)	7.3(7.2-7.4)	5.5(5.4-5.6)	

Table S5. The frequency of self-reported history of disease in the participants based on a physician diagnosis.

			**Age groups**			
**Coronary Artery Disease**	yes	**Gender**	**60 – 69 years N = 787 (%)**	**70 – 79 years N = 353 (%)**	**≥80 years N = 111 (%)**	**Total N=1251**
		Female	10.1(10.0-10.2)	21.0(20.8-21.1)	10.5(10.3-10.7)	18.4(18.3-18.4)
		Male	26.9(26.8-27.0)	20.9(20.7-21.0)	14.8(14.6-15.0)	
**Cerebrovascular disease**	yes	Female	4.6(4.5-4.6)	6.2(6.1-6.3)	8.7(8.6-8.9)	6.4(6.3-6.4)
		Male	5.3(5.3-5.4)	9.0(8.9-9.1)	11.1(10.9-11.2)	
**BPH**	yes	Male	44.2(44.0-44.3)	46.2(46.0-46.4)	54.9(54.6-55.1)	49.3(49.3-49.4)
**Hyperlipidemia**	yes	Female	43.4(43.3-43.5)	54.5(54.3-54.7)	38.5(38.3-38.8)	40.6(40.5-40.6)
		Male	37.7(37.6-37.8)	34.4(34.3-34.6)	25.9(25.6-26.1)	
**Dementia**	yes	Female	20.9(20.8-21.0)	27.8(27.6-27.9)	42.1(41.8-42.3)	22.5(22.4-22.6)

		Male	15.8(15.7-15.9)	22.0(21.9-22.1)	29.6(29.3-29.8)	
**Seizure**	yes	Female	3.5(3.4-3.5)	2.8(2.7-2.8)	3.5(3.4-3.6)	2.9(2.8-2.9)
		Male	1.1(1.1-1.2)	4.5(4.4-4.5)	3.7(3.5-3.8)	
**Urinary tract infection**	yes	Female	28.2(28.1-28.3)	31.8(31.6-31.9)	36.8(36.5-37.1)	24.0(24.0-24.1)
		Male	15.2(15.1-15.3)	19.2(19.0-19.3)	25.9(25.6-26.1)	
**Digestive Problem**	yes	Female	28.2(28.1-28.3)	36.9(36.7-37.0)	35.0(34.8-35.3)	31.5(31.4-31.5)
		Male	30.2(30.1-30.3)	28.8(28.6-28.9)	42.5(42.3-42.8)	
**Pulmonary diseases**	yes	Female	13.6(13.6-13.7)	16.4(16.3-16.5)	19.2(19.0-19.5)	13.5(13.4-13.5)
		Male	11.3(11.3-11.4)	9.6(9.5-9.6)	18.5(18.2-18.7)	
**Ever Asthma**	yes	Female	10.1(10.0-10.2)	9.0(8.9-9.1)	7.0(6.8-7.1)	8.7(8.7-8.8)
		Male	7.7(7.7-7.8)	7.3(7.2-7.4)	11.1(10.9-11.2)	
**Osteoarthritis**	yes	Female	53.6(53.5-53.7)	62.5(62.3-62.6)	73.6(73.4-73.9)	46.1(46.0-46.1)
		Male	28.4(28.3-28.5)	37.8(37.6-38.0)	46.2(46.0-46.5)	
**Osteoporosis**	yes	Female	49.8(49.7-49.9)	61.9(61.7-62.0)	71.9(71.6-72.1)	40.5(40.4-40.5)
		Male	20.3(20.2-20.4)	31.0(30.9-31.2)	31.4(31.2-31.7)	
**Diabetes**	yes	Female	23.3(23.3-23.4)	31.2(31.1-31.3)	29.8(29.5-30.0)	23.9(23.9-24.0)
		Male	23.3(23.2-23.4)	22.5(22.4-22.7)	11.1(10.9-11.2)	
**Thyroid**	yes	Female	15.6(15.6-15.7)	8.5(8.4-8.6)	5.2(5.1-5.3)	9.14(9.11-9.18)
		Male	5.6(5.6-5.7)	8.4(8.3-8.5)	1.8(1.7-1.9)	
**Cataract**	yes	Female	24.6(24.5-24.6)	54.8(54.7-55.0)	66.6(66.4-66.9)	37.2(37.1-37.2)
		Male	25.1(24.9-25.2)	48.9(48.7-49.0)	54.5(54.3-54.8)	
**Glaucoma**	yes	Female	2.2(2.1-2.2)	5.6(5.6-5.7)	5.2(5.1-5.3)	3.6(3.6-3.7)
		Male	3.2(3.2-3.3)	3.9(3.8-4.0)	5.5(5.4-5.6)	
**Cancer**	yes	Female	4.1(4.1-4.2)	5.1(5.0-5.1)	7.0(6.8-7.1)	4.74(4.71-4.76)
		Male	5.0(5.0-5.1)	3.3(3.3-3.4)	5.5(5.4-5.6)	
**Depression**	yes	Female	37.0(36.9-37.1)	41.4(41.3-41.6)	57.8(57.6-58.1)	34.4(34.4-34.5)

		Male	23.9(23.8-24.0)	31.0(30.9-31.2)	38.8(38.6-39.1)	

Table S6. The frequency of self-reported multi morbidity in the participants based on a physician diagnosis.

			Age groups			
		**Gender**	**60 – 69 years N = 787 (%)**	**70 – 79 years N = 353 (%)**	**≥80 years N = 111 (%)**	**Total N=1251**
**Multi morbidity**	No diseases	Female	11.0(10.9-11.1)	2.8(2.7-2.8)	5.2(5.1-5.3)	9.66(9.62-9.69)
		Male	14.0(13.9-14.1)	8.4(8.3-8.5)	5.5(5.4-5.6)	
	one to two diseases	Female	33.1(33.0-33.2)	23.8(23.7-24.0)	12.2(12.0-12.4)	32.8(32.7-32.8)
		Male	39.8(39.7-39.9)	36.7(36.5-36.8)	31.4(31.2-31.7)	
	three disease or more	Female	55.8(55.7-55.9)	73.2(73.1-73.4)	82.4(82.2-82.6)	57.5(57.4-57.5)

Table S7. Visual and hearing status in age and sex groups

			Age groups			
**Glasses**		**Gender**	**60 – 69 years N = 787 (%)**	**70 – 79 years N = 353 (%)**	**≥80 years N = 111 (%)**	**Total N = 1251**
	no	Female	35.3(35.2-35.4)	35.2(35.0-35.3)	38.5(38.3-38.8)	35.4(35.3-35.5)
		Male	36.2(36.1-36.3)	30.5(30.3-30.6)	40.7(40.4-41.0)	
	yes	Female	64.6(64.5-64.7)	64.7(64.6-64.9)	61.4(61.1-61.6)	64.5(64.4-64.6)
		Male	63.7(63.6-63.8)	69.4(69.3-69.6)	59.2(58.9-59.5)	
**Eyesight Status**	Good	Female	55.6(55.5-55.7)	46.5(46.4-46.7)	31.5(31.3-31.8)	52.2(52.2-52.3)
		Male	54.4(54.3-54.6)	55.3(55.2-55.5)	53.7(53.4-53.9)	
	mild impairment	Female	24.0(23.9-24.1)	30.6(305-30.8)	31.5(31.3-31.8)	26.2(26.1-26.2)
		Male	25.4(25.3-25.5)	28.8(28.6-28.9)	18.5(18.2-18.7)	
	moderate impairment	Female	20.0(20.0-20.1)	22.7(22.5-22.8)	35.0(34.8-35.3)	21.2(21.1-21.2)
		Male	20.0(19.9-20.1)	15.2(15.1-15.3)	27.7(27.5-28.0)	
	Blind	Female	0.22(0.21-0.23)	0	1.7(1.6-1.8)	0.25(0.25-0.26)
		Male	0	0.56(0.54-0.58)	0	
**Hearing Aid**	no	Female	94.0(93.9-94.0)	92.0(91.9-92.1)	84.2(84.0-84.4)	91.4(91.3-91.4)
		Male	93.1(93.0-93.1)	88.1(88.0-88.2)	85.1(84.9-85.3)	
	yes	Female	5.9(5.9-6.0)	7.9(7.8-8.0)	15.7(15.5-15.9)	8.5(8.5-8.6)
		Male	6.8(6.8-6.9)	11.8(11.7-11.9)	14.8(14.6-15.0)	
**Hearing Status**	Good	Female	78.3(78.2-78.4)	71.0(70.8-71.1)	52.6(52.3-52.9)	71.8(71.8-71.9)
		Male	75.7(75.6-75.8)	67.7(67.6-67.9)	55.5(55.2-55.8)	
	Mild impairment	Female	14.3(14.2-14.4)	18.7(18.6-18.8)	24.5(24.3-24.8)	16.5(16.5-16.6)
		Male	14.6(14.5-14.7)	20.9(20.7-21.0)	12.9(12.7-13.1)	
	Moderate	Female	6.1(6.1-6.2)	9.0(8.9-9.1)	15.7(15.5-15.9)	9.5(9.4-9.5)

	impairment	Male	80.(8.0-8.1)	8.4(8.3-8.5)	27.7(27.5-28.0)	
	Severe deafness	Female	1.1(1.0-1.1)	1.13(1.10-1.17)	7.0(6.8-7.1)	2.02(2.00-2.04)
		Male	1.4(1.4-1.5)	2.8(2.7-2.8)	3.7(3.5-3.8)	

Table S8. Teeth and Eating Ability status in age and sex groups

			**Age groups**			
**Dentures**		**Gender**	**60 – 69 years N = 787 (%)**	**70 – 79 years N = 353 (%)**	**≥80 years N = 111 (%)**	**Total N = 1251**

	no	Female	46.1(46.0-46.2)	28.9(28.8-29.1)	28.0(27.8-28.3)	39.7(39.7-39.8)
		Male	51.4(51.3-51.6)	27.6(27.5-27.8)	20.3(20.1-20.6)	
	yes	Female	53.8(53.7-53.9)	71.0(70.8-71.1)	71.9(71.6-72.1)	60.2(60.1-60.2)
		Male	48.5(48.3-48.6)	72.3(72.1-72.4)	79.6(79.3-79.8)	
**Teeth**	no any teeth in mouth	Female	25.7(25.6-25.8)	40.2(40.0-40.3)	49.1(48.8-49.4)	33.3(33.2-33.3)
		Male	22.6(22.5-22.7)	44.3(44.1-44.4)	60.3(60.0-60.6)	
	1-9 teeth in mouth	Female	12.4(12.3-12.4)	18.3(18.2-18.5)	19.2(19.0-19.5)	13.84(13.80-13.88)
		Male	14.1(14.1-14.2)	8.5(8.4-8.6)	15.0(14.8-15.3)	
	10-19 teeth in mouth	Female	20.8(20.7-20.9)	17.2(17.1-17.3)	8.7(8.6-8.9)	19.8(19.8-19.9)
		Male	21.1(21.0-21.2)	27.8(27.6-27.9)	9.4(9.2-9.6)	
	20 or more teeth in mouth	Female	41.0(40.9-41.1)	24.1(24.0-24.2)	22.8(22.5-23.0)	32.9(32.8-33.0)
		Male	41.9(41.8-42.1)	19.3(19.1-19.4)	15.0(14.8-15.3)	
**Eating Ability**	no	Female	9.7(9.7-9.8)	12.5(12.4-12.6)	24.5(24.3-24.8)	9.6(9.6-9.7)
		Male	6.9(6.9-7.0)	4.5(4.5-4.6)	11.1(10.9-11.2)	
	yes	Female	90.2(90.1-90.2)	87.4(87.3-87.5)	75.4(75.1-75.6)	90.3(90.2-90.3)
		Male	93.0(92.9-93.0)	95.4(95.3-95.4)	88.8(88.7-89.0)	

Table S9. Sleep status in age and sex groups

			**Age groups**			
**Good sleep**		**gender**	**60 – 69 years N = 787 (%)**	**70 – 79 years N = 353 (%)**	**≥80 years N = 111 (%)**	**Total N = 1251**
	no	Female	35.6(35.6-35.7)	44.8(44.7-45.0)	40.3(40.0-40.6)	32.7(32.6-32.8)
		Male	26.0(25.9-26.1)	28.5(28.4-28.7)	24.0(23.8-24.3)	

	yes	Female	64.3(64.2-64.3)	55.1(54.9-55.2)	59.6(59.3-59.9)	67.2(67.1-67.3)
		Male	73.9(73.8-74.0)	71.4(71.2-71.5)	75.9(75.6-76.1)	

Table S10. The urine and stool continence status of the participants in age and sex groups

			**Age groups**			
**Sphincter control**		**Gender**	**60 – 69 years N = 787 (%)**	**70 – 79 years N = 353 (%)**	**≥80 years N = 111 (%)**	**Total N = 1251**
**Urine continence**	**not have continence**	Female	2.64(2.61-2.68)	3.4(3.3-3.4)	19.2(19.0-19.5)	5.3(5.3-5.4)
		Male	1.4(1.4-1.5)	3.9(3.8-4.0)	7.4(7.2-7.5)	
	**sometimes not have control**	Female	22.0(21.9-22.1)	33.5(33.3-33.6)	31.5(31.3-31.8)	23.0(23.0-23.1)
		Male	14.0(13.9-14.1)	24.8(24.7-24.9)	29.6(29.3-29.8)	
		Female	75.2(75.1-75.3)	63.0(62.9-63.2)	49.1(48.8-49.4)	

	**have control**					71.5(71.4-71.5)
		Male	84.4(84.3-84.5)	71.1(71.0-71.3)	62.9(62.6-63.2)	
**Stool continence**	**not have continence**	Female	1.32(1.30-1.34)	3.4(3.3-3.4)	10.5(10.3-10.7)	3.1(3.1-3.2)
		Male	0.8(0.8-0.9)	2.8(2.7-2.8)	14.8(14.6-15.0)	
	**sometimes not have control**	Female	3.5(3.4-3.5)	6.2(6.1-6.3)	14.0(13.8-14.2)	4.8(4.7-4.8)
		Male	2.0(2.0-2.1)	4.5(4.4-4.5)	11.1(10.9-11.2)	
	**have control**	Female	95.14(95.10-95.18)	90.3(90.2-90.4)	75.4(75.1-75.6)	91.9(91.9-92.0)
		Male	97.0(96.9-97.0)	92.6(92.5-92.7)	74.0(73.8-74.3)	
